# A systematic study of the production of Monacolin K by solid state fermentation of *Monascus ruber*

**DOI:** 10.1186/s13568-022-01368-z

**Published:** 2022-03-03

**Authors:** Xiuhe Liu, Aonan Sun, Qing Li, Yamin Du, Tao Zhao

**Affiliations:** School of Food Science and Engineering, Qilu University of Technology (Shandong Academy of Sciences), Jinan, 250353 People’s Republic of China

**Keywords:** *Monascus*, Monacolin K, Solid state fermentation, Statistical parameter analysis

## Abstract

Monacolin K is one of the bioactive substances produced by *Monascus ruber* during fermentation. The multi-factors and their interactions on the effect of solid-state fermentation of *Monascus* for high yield of monacolin K were attractive to industrial production. A detailed study of 7 single-factor experiments and a series of experiments with Plackette-Burman and Box-Benhnken design, data fitting and modeling, and analyzing the visual 3D response surface plots for investigation of the key factors for Monacolin K production. The results showed that initial moisture (50 ~ 55%) and bran content (4.5 ~ 5.5%) as the key factors of transport for nutrients and oxygen during the solid-state fermentation (SSF) process of *Monascus*. Under the optimal conditions, a temperature shifting of the SSF with a higher Monacolin K yield of 14.53 ± 0.16 mg·g^− 1^ compared with the content of monacolin K in the commercially available functional red yeast rice of 8 mg g^− 1^.

**Supplementary Information:**

The online version contains supplementary material available at 10.1186/s13568-022-01368-z.

## Key points

- A systematic study for Monacolin K produced by SSF of *Monascus ruber.*
- Initial moisture and bran content are the key factors for the SSF of Monascus.
- A proper bran content could suppress the influence of excessive media amount.

**Supplementary Information:**

The online version contains supplementary material available at 10.1186/s13568-022-01368-z.

## Introduction

Monacolin K (MK) is the principal active substance in *Monascus ruber* fermentation products (e.g. red yeast rice). MK is effective in reducing cholesterol levels in humans and has been widely used as a lipid-lowering drug (Lovastatin). The bioactivity of MK conducted in-depth research around the world, such as preventing colon cancer, acute myeloid leukemia, and neurological disorders in addition to lowering blood lipid levels (Xiong et al. 2019). Moreover, red yeast rice has proven to be a functional food for effectively controlling hypercholesterolemia (Ezhov et al. 2020). Therefore, how to obtain a higher yield of MK in the fermentation process present meaningful for the biomedicine and functional foods fields.

Generally, *Monascus* strains, fermentation methods, and fermentation conditions have special effects on MK yield. Mutagenesis of *Monascus* is performed by physical or chemical methods, such as UV irradiation (Sun et al. 2011), γ-ray (Suh et al. 2007), and chemical agent treatment (Zhang et al. 2017) to obtain strains with a high yield of MK. Solid-state fermentation (SSF) is perfectible compared to submerged fermentation (SmF) due to their more rapid rate of cell growth and the better fluidity and permeability of the cell membrane (Mohan-Kumari et al. 2012; Zhang et al. 2015). In a recent study, Wen and their co-authors summarized that the main parameters affecting the MK production of *Monascus* fermentation products contain an initial moisture content of the solid matrix, initial pH of the medium, inoculum size, culture temperature, and fermentation time (Wen et al. 2020). Some interesting works have been proposed for optimization of the SSF medium components of *Monascus* (Lee et al. 2012; Chang et al. 2002). Some works focused on the comparison of MK yield by *Monascus* fermentation using different matrixes (Handa et al. 2019; Subhagar et al. 2009; Suraiya et al. 2018). Some works focused on the comparison of MK yield by *Monascus* fermentation under different parameters (Dikshit et al. 2016; Panda et al. 2010; Lu et al. 2013). However, a detailed study of the multi-factors and their interactions on the effect of SSF of *Monascus* for high yield of monacolin K was left unexplored.

In this study, we firstly screened the high-yield monacolin K strain (*Monascus ruber* K 10,403), and then the seed age, initial moisture, carbon sources and nitrogen sources, initial pH, bran content, variable temperature, inoculation amount, size of the rice grain, media amount, and fermentation time were set as single-factor experiments, respectively. Plackette-Burman design was then applied to investigate the significant variables of the 7 single factors for Monacolin K production. The Plackette-Burman results showed the initial moisture, bran content, and media amount are the three key factors for MK production. Further, the Box-Benhnken central grouping and the response surface methodology (RSM) method were applied to obtain the optimal condition and study their interactions of bran content, media amount, initial moisture on the production of monacolin K.

## Materials and methods

### Materials

*Monascus rubber* (K 10,403) is supplied by Shandong Zhonghui Biotechnology Co., Ltd (Binzhou, Shandong, China). The Monacolin K standard products were purchased from Sigma-Aldrich (Shanghai, China). Rice, bran, potatoes, and soybean meal were purchased from local supermarkets (Jinan, Shandong, China). Glucose, corn syrup, maltose, glycerol, sucrose, yeast powder, and agar powder were purchased from Beijing Obstar Technology Co., Ltd. (Beijing, China). Sodium hydroxide, ethanol (analytical pure), and methanol (chromatography pure) were purchased from Sinopharm Chemical Reagent Co., Ltd. (Shanghai, China). Lactic acid was purchased from Henan Xinghan Biological Technology Co., Ltd. (Puyang City, Henan Province, China). Sodium nitrate and ammonium nitrate were purchased from Tianjin Hengxing Chemical Reagent Manufacturing Co., Ltd (Tianjin, China). Phosphoric acid was purchased from Laiyang Economic and Technological Development Zone Fine Chemical Co., Ltd (Yantai City, Shandong Province, China). Peptone was purchased from Beijing Shuangxuan Microbial Culture Medium Manufacturing Factory (Beijing, China).

### Culture media and methods

The seed media consisted of 200 g L^− 1^ of potato, 20 g L^− 1^ of glucose, the seed media was sterilized at 121℃ for 20 min. The SSF medium consisted of rice (500 g L^− 1^), bran (50 g L^− 1^), glucose (35 g L^− 1^), peptone (15 g L^− 1^) with a pH value of 5.0. The SSF medium was sterilized at 115℃ for 15 min.

The *Monascus* spores were scraped off from the test-tube slant cultivation and then diluted to a concentration of 5.7 × 10^3^ spores mL^− 1^ as the spore suspension. 15% of the spore suspension was inoculated into a 200 mL seed medium, and then placed in a shaker incubator at 30℃ at a speed of 160 rpm for different times (12 ~ 60 h). The seeds were then inoculated into SSF medium and incubated at a low temperature for 3 days, and then at a high temperature for 15 days.

### Extraction and analysis of Monacolin K

After SSF for 18 days, the red yeast rice was dried in an oven at 50℃ until constant weight. The dried red yeast rice was then put into a grinder to crush for 1 min and then passed through a 40-mesh sieve. The extraction method refers to the relevant literature with minor modifications (Zhou et al. 2014; Theunis et al. 2017): 20 ~ 30 mg of the treated powders with 30 mL of 75% ethanol were added into a 50 mL volumetric flask, and then ultrasonic extracted twice for 30 min at room temperature. The extraction was transferred to a 50 mL centrifuge tube and centrifuged at 4000 r min^− 1^ for 10 min. The supernatant was collected and filtered through a 0.45 μm filterer membrane. The concentration of the extracted Monacolin K was determined by high-performance liquid chromatography (HPLC, LC-15 C, Shimadzu, Tokyo, Japan) with a kromasil C_18_ column (250 mm×4.6 mm, 5 μm) and a mobile phase of methanol/water/phosphoric acid (385/115/0.14, v/v/v), the flow rate of 1 mL min^− 1^, column temperature of 20 ~ 25℃ and injection volume of 20 µL were used. The UV detection wavelength was set at 238 nm.

### Single factor experiments for solid state fermentation

#### Optimization of initial moisture

Initial moisture plays an important role in the nutrient utilization and physiological activities of *Monascus* in SSF. The effect of initial moisture on the yield of monacolin K was investigated by the solid-fermentation medium of different initial moisture (20%, 30%, 40%, 50%, and 60%). The water content was determined as follows: the weighing bottle and cap were dried in an oven of 95 ~ 105℃ for 1 h, and then placed in a drying basin to cool for 0.5 h. 10 g solid state fermentation medium was added into the weighing bottle with a sample thickness of 5 mm. The sample weighing bottle was placed in an oven at 95 ~ 105℃ for drying until constant weight (< 2 mg) before use. The initial moisture was calculated by the following formula (1):

1 $${\text{X}} = {{\left( {{\text{m}}_{{1}} - {\text{m}}_{{2}} } \right)} \mathord{\left/ {\vphantom {{\left( {{\text{m}}_{{1}} - {\text{m}}_{{2}} } \right)} {\left( {{\text{m}}_{{1}} - {\text{m}}_{{3}} } \right)}}} \right. \kern-\nulldelimiterspace} {\left( {{\text{m}}_{{1}} - {\text{m}}_{{3}} } \right)}} \times { 1}00\%$$

Here m_1_ is the weight of sample weighing bottle before drying (g); m_2_ is the weight of the sample weighing bottle after drying (g); m_3_ is the weight of the empty weighing bottle (g).

#### Optimization of initial pH

pH has a significant effect on the catalytic process of many enzymes and the transport of various components of the cell membrane, thus affecting the use of substrates by fungi. The pH value of the SSF medium was adjusted to 4.0, 5.0, 6.0, 7.0, 8.0, 9.0 by lactic acid, respectively. The pH value was measured as follows: 10 g (± 0.01) solid medium was accurately weighed in a beaker (250 mL), and then 100 mL of double distilled water was added and stirred for 30 min. The above solution was centrifuged at 4000 r min^− 1^ for 5 min, 50 mL of the clear supernatant was transferred into a beaker (100 mL) and measured 3 times by a pH meter (PHS-3 C, REX, Shanghai, China).

#### Optimization of different carbon sources and nitrogen sources

Glucose, maltose, sucrose, glycerol, and soluble starch were set as the carbon sources for solid-state fermentation medium (the content of 2 ~ 4%), respectively. Peptone, corn steep liquor, yeast powder, ammonium nitrate and sodium nitrate were set as the nitrogen sources for solid-state fermentation medium (the content of 0.5 ~ 2.5%), respectively. The SSF medium without additional carbon and nitrogen source was used as the control groups.

#### Optimization of bran content and rice grains size

The SSF medium contained 0, 2.5, 5, 7.5, 10, and 12.5% bran were used to study the influence of bran content on the production of Monacolin K, respectively. The SSF medium contained rice grains that were prepared through no-sieve, 12 mesh, 14 mesh, and 20 mesh of sieve for the investigation of the influence of rice grain size on the production of monacolin K.

#### Optimization of inoculation and media amount

The inoculation amount of *Monascus* strains was cultured for 4, 8, 12, 16, and 20%, respectively. The basic medium with media amounts of 30, 45, 60, 75, and 90 g was added to a flask (500 mL) for solid-state fermentation, respectively. The influence of different inoculation and media amount on the yield of monacolin K was investigated by the content of Monacolin K in red yeast rice after fermentation.

#### Optimization of fermentation time and temperature

Culture time and temperature are critical for the growth of filamentous fungi and the production of secondary metabolites. The SSF medium was used for shake flask fermentation, and the samples were taken every 24 h to determine the content of monacolin K in red yeast rice for the investigation of different fermentation times on the production of Monacolin K. The constant and variable temperature fermentation were performed by using SSF medium as the basic medium. For the constant temperature fermentation, the temperatures were set as 26, 28, 30, and 32 ℃, respectively. For the variable temperature fermentation, the temperature was performed at 30 ℃ in the first 3 days, and then 26 ℃ for15 days.

### Statistical parameter analysis

Plackette-Burman design was applied to investigate the significant variables responsible for Monacolin K production based on the results of the single-factor experiments. Each variable was tested at two levels, high level (+) and low levels (−), and four variables were screened by conducting 12 experiments. All data were analyzed through OriginPro 2016 and Design-Expert 12 software. The interaction effects were investigated according to Box-Benhnken central grouping method and response surface methodology.

## Results

### HPLC detection method for Monacolin K content

Additional file 1: Figure S1 showed the HPLC chromatogram chart of Monacolin K, the results indicated the established HPLC method has a good chromatographic resolution for acidic and lovastatin Monacolin K with less matrix interference. The method showed a good linear range from 5 mg L^-1^ to 100 mg L^-1^ with a regression coefficient of 0.998 (Additional file 1: Figure S2). The red yeast rice spiked with lovastatin Monacolin K at six levels (40 ~ 240 mg L^-1^) were analyzed by the proposed method. The recovery ranges from 95.58 to 103.52%, and the relative standard deviation (RSD) of 0.04% (Additional file 1: Table S1).

## Single-factor experiments for solid-state fermentation

### Influences of initial moisture and pH on the production of Monacolin K

**Fig. 1:**
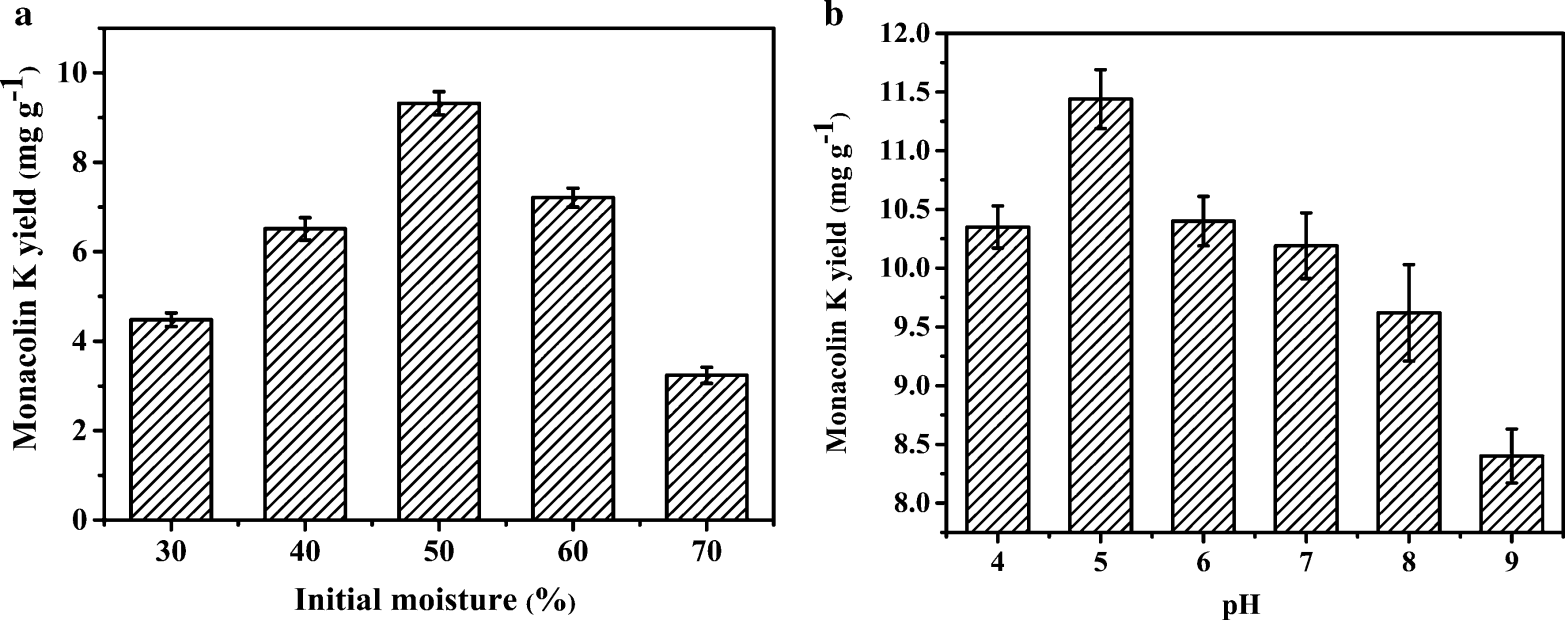
Influences of initial moisture (**a**) and pH (**b**) on the production of Monacolin K

The results in Fig. 1a indicated the insufficient initial moisture (< 30%) inhibited the growth of *Monascus*, which would result in a low content of Monacolin K (4.34 mg g^− 1^). As the initial moisture increased to 50%, the yield of Monacolin K reached the maximum value of 9.24 mg g^− 1^. However, the too high initial moisture (> 70%) would depress the yield of Monacolin K (3.29 mg g^− 1^). The results in Fig. 1b indicated that weak acidic condition was favorable to the production of Monacolin K, while the yield of Monacolin K was significantly inhibited in weak alkali conditions. The optimal pH value of 5.0 for solid-state fermentation production of Monacolin K.

### Influences of carbon and nitrogen sources on the production of Monacolin K

**Fig. 2:**
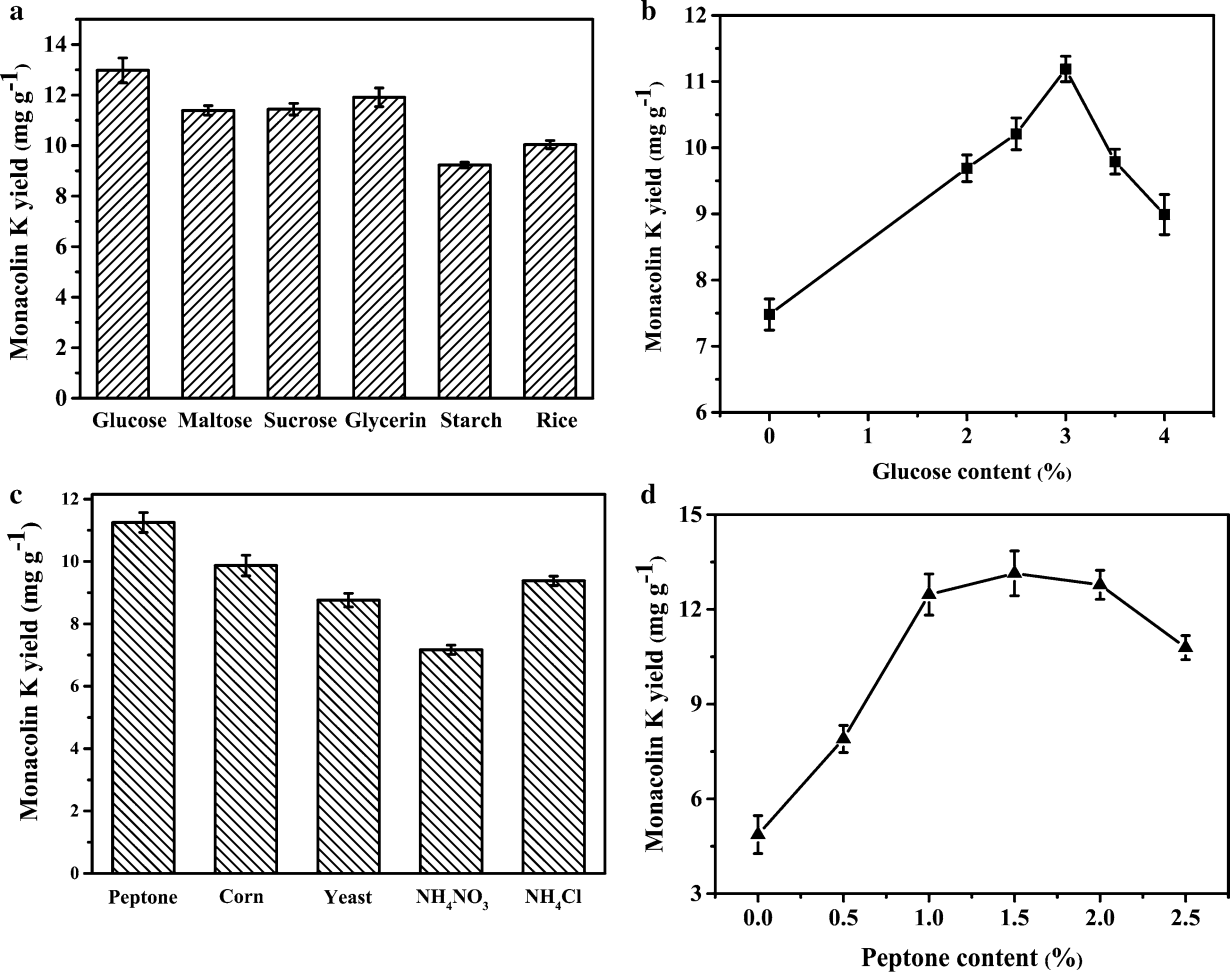
Influences of different carbon sources (**a**) and glucose content (**b**), nitrogen sources (**c**) and peptone content (**d**) on the yield of Monacolin K

The growth and metabolism of microorganisms are inseparable from carbon and nitrogen sources. The results in Fig. 2a showed the higher yield of Monacolin K was obtained by using glucose, the low yield of Monacolin K was obtained by using starch. The production of Monacolin K is relatively high by using glycerin as a carbon source. The reason may be glycerol has a certain influence on the synthesis of Monacolin K-related genes (Zhang et al. 2019). Figure 2b showed the highest Monacolin K production of 11.19 mg·g^− 1^ under the optimal glucose content of 3%. The results in Fig. 2c showed the highest yield of Monacolin K was obtained by using peptone as an additional nitrogen source, the optimal peptone content was 1.5% with a maximum yield of Monacolin K of 13.14 mg g^− 1^ (Fig. 2d).

### Influences of bran content and rice grain size on the production of Monacolin K

**Fig. 3:**
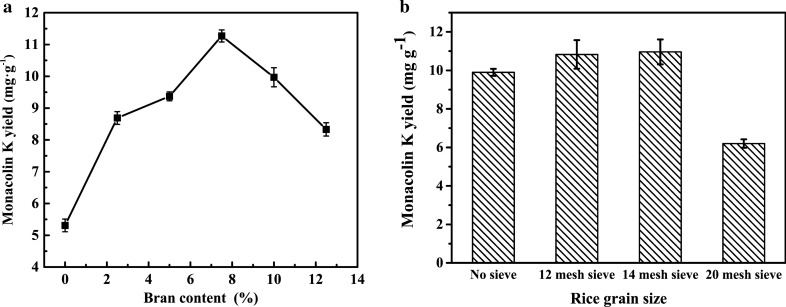
Influences of different bran content (a) and rice grain size (b) on Monacolin K

The results in Fig. 3a showed the production of Monacolin K increased with the increasing of bran content and the maximum yield was obtained by the addition of 7% bran content. The size of rice grains affects the contact area between strain and rice, which is an important factor for the absorption and utilization of nutrients by *Monascus*. Figure 3b depicted the influences of rice grain sizes on the production of Monacolin K. The production of Monacolin K increased with the rice grain sizes decreased and the optimal rice grain sizes of 14 mesh sieve with the highest yield of 10.96 mg g^− 1^. However, with the size of the rice particles continuing to decrease to 20 mesh, the medium stuck to clumps, which would hinder the absorption of oxygen by *Monascus*, resulting in a lower production of Monacolin K.

### Influences of inoculation and media amount on the production of Monacolin K

**Fig. 4:**
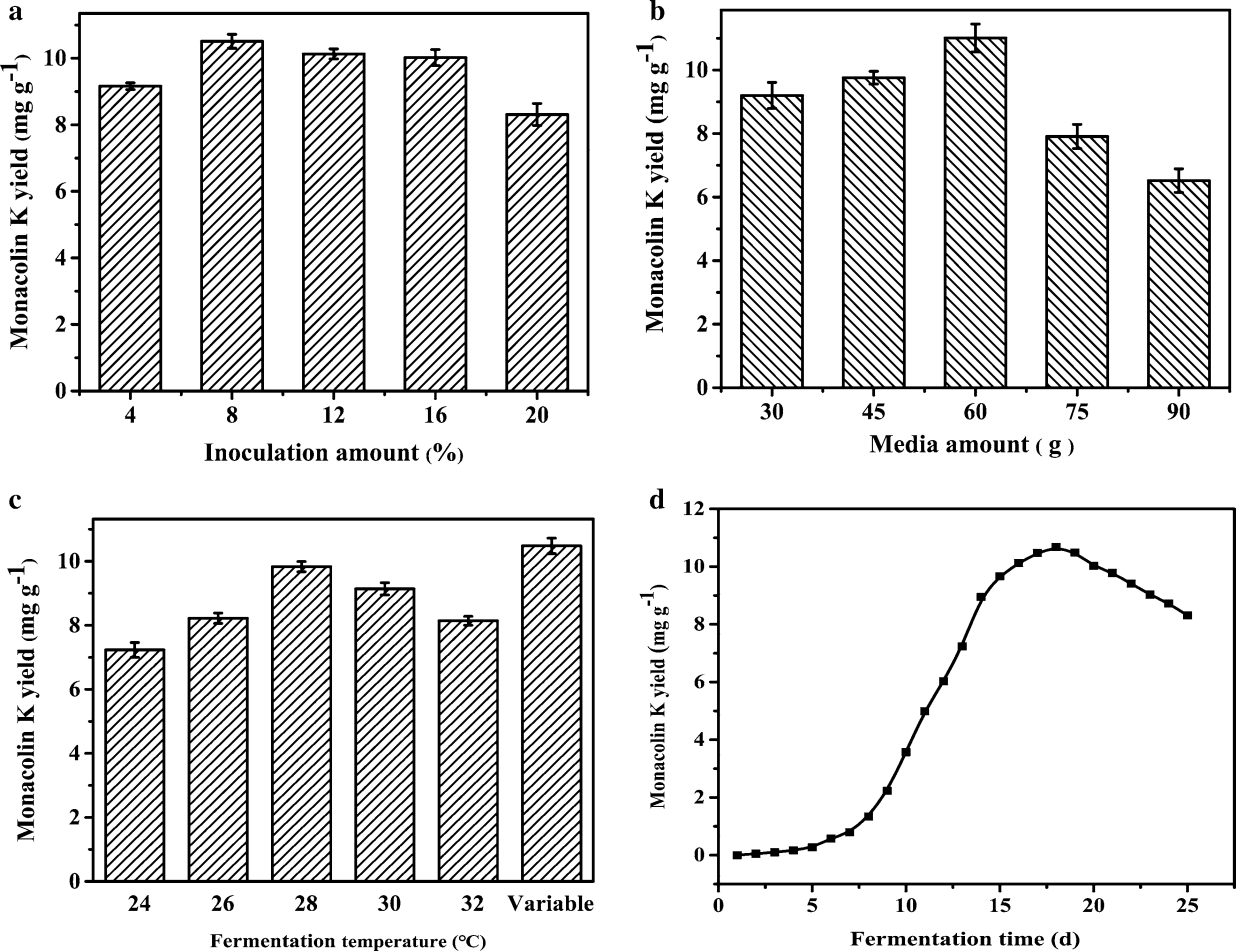
Influences of inoculation amount (**a**), media amount (**b**), fermentation temperature (**c**) and time (**d**) on the yield of Monacolin K

The production of Monacolin K is directly related to the inoculation amount of *Monascus*. Figure 4a showed the production of Monacolin K increased significantly with the increase of inoculum amount from 4 to 8%. The optimal inoculation amount was 8% with a maximum yield of Monacolin K of 10.51 mg g^− 1^. However, lower production of Monacolin K was observed with the increase of inoculation amount from 12 ~ 20%. The reason may be the proliferation of *Monascus* cells leads to insufficient nutrients and oxygen supply in the culture medium, which hinder the SSF process. The effects of media amount on the production of Monacolin K by *Monascus* was investigated by using different media amounts. The results in Fig. 4b showed the optimal media amount was 60 g with a maximum Monacolin K production of 11.01 mg·g^− 1^. However, when the media amount was greater than 60 g, the production of Monacolin K decreased obviously with the increase of media amount.

### Influences of fermentation temperature and time on the production of Monacolin K

Figure 4c depicted the influences of the different fermentation temperatures on the production of Monacolin K. The results indicated variable temperature fermentation is the optimal conditions among the temperature of 24, 26, 28, 30, and 32 °C. The results in Fig. 4d indicated Monacolin K was produced and accumulated slowly in the early stage of fermentation. The Monacolin K production increased logarithmically after 8 days of fermentation. The maximum production of Monacolin K was obtained after 18 days of fermentation, and then the Monacolin K content gradually decreased as the fermentation time continued to increase. Therefore, the most suitable fermentation time for the production of Monacolin K by *Monascus* was 18 days with a maximum yield of 10.68 mg·g^− 1^.

## Significant factors and interaction analysis

### Plackett-Burman Screening for significant factors

Based on single-factor experiments, the significant factor screening of initial moisture (X_1_), glucose content (X_2_), peptone content (X_3_), bran content (X_4_), initial pH (X_5_), media amount (X_6_), and inoculation amount (X_7_) on the production of Monacolin K were identified by Plackett-Burman design with 4 dummy variables for error analysis (X_8_, X_9_, X_10_, X_11_). The Plackett-Burman design was shown in Additional file 1: Table S2.

The results of the quadratic regression model variance analysis were shown in Table 1. A polynomial regression equation was obtained by the Design Expert 12 software analysis (2):

2 $$ \begin{aligned}{\text{Y}}\, &= \,{9}.0{3} + {1}.{4}0*{\text{X}}_{{1}} + 0.{8}0*{\text{X}}_{{2}} + 0.{19}*{\text{X}}_{{3}} \\ &\quad- {1}.{27}*{\text{X}}_{{4}} + 0.{36}*{\text{X}}_{{5}} - {1}.0{4}*{\text{X}}_{{6}} + 0.{42}*{\text{X}}_{{7}}\end{aligned} $$

Where Y is the yield of Monacolin K (mg g^− 1^); X_1_ is the initial moisture (%); X_2_ is the glucose content (%); X_3_ is the peptone content (%); X_4_ is the bran content (%); X_5_ is the initial pH; X_6_ is the media amount (g) and X_7_ is the inoculation amount (%).

**Table 1:** Plackett-Burman experiment analysis results of single-factor experiments

Source	Sum of squares	Degree of freedom	Mean squares	F-value	Prob > F
Model	67.66	7	9.67	15.75	0.0091**
X_1_	23.49	1	23.49	38.27	0.0035**
X_2_	7.76	1	7.76	12.64	0.0237*
X_3_	0.45	1	0.45	0.74	0.4390
X_4_	19.33	1	19.33	31.49	0.0050**
X_5_	1.53	1	1.53	2.5	0.1891
X_6_	13.00	1	13.00	21.18	0.0100**
X_7_	2.09	1	2.09	3.14	0.1381

Table 1 showed the regression model was very significant (P < 0.01), the lack-of-fit term was not significant (P > 0.05), and the correlation coefficient R^2^ was 0.9650. The results indicated that the initial moisture (X_1_), the bran content (X_4_), and the media amount (X_6_) were significant factors (P < 0.01); While, the glucose content (X_2_), peptone content (X_3_), initial pH (X_5_), and inoculation amount (X_7_) were not significant.

## Box-Benhnken experiment analysis

Box-Benhnken central grouping method was used to design three factors and three levels test based on the Plackett-Burman Screening results. The Box-Benhnken experiment design method was shown in Additional file 1: Table S3, the non-significant factors were fixed at 3% of glucose content, 1.5% of peptone content, initial pH of 5.0 and 8% of inoculation amount.

The results of the analysis of variance for the regression model of Monacolin K produced by *Monascus* were shown in Table 2. A quadratic polynomial fitting regression equation was obtained according to the relevant data of the quadratic regression fitting analysis (3):

3 $$ \begin{aligned}{\text{Y}} & = {14}.{4}0 + 0.{85}*{\text{ A}} - 0.{26}*{\text{B}} + {1}.{5}0*{\text{C}} \\ &\quad+ {\text{AB}} + 0.{55}*{\text{AC}} + 0.{62}*{\text{BC}} - {3}.{26}\\ &\quad*{\text{A}}^{{2}} - 0.{88}*{\text{B}}^{{2}} - {2}.{8}0*{\text{C}}^{{2}}\end{aligned} $$

Where Y is the yield of Monacolin K (mg g^− 1^); A is the bran content (%); B is the media amount (g); C is the initial moisture (%).

The model F-value of 95.79 implies the model is significant. The p-values less than 0.05 indicate model terms are significant. In this case, A, C, AB, AC, BC, A^2^, B^2^, and C^2^ are significant model terms. The influence order of the three significant factors is initial moisture (C) > bran content (A) > media amount (B), the interaction strength order is AB > BC > AC. The correlation coefficient (R^2^ = 0.992), the Predicted R^2^ of 0.928 is in reasonable agreement with the Adjusted R^2^ of 0.982, which indicated that the regression model had a low error, high reliability, and fitting degree.

**Table 2:** Box-Benhnken experiment results for significant factors

Source	Coefficient	Sum of squares	df	Mean squares	F-value	p-value
Model	14.40	117.92	9	13.10	95.79	< 0.0001**
A	0.85	5.73	1	5.73	41.88	0.0003**
B	− 0.26	0.53	1	0.53	3.88	0.0896
C	1.50	17.97	1	17.97	131.38	< 0.0001**
AB	0.82	2.72	1	2.72	19.90	0.0029**
AC	0.55	1.22	1	1.22	8.93	0.0203*
BC	0.62	1.54	1	1.54	11.24	0.0122*
A^2^	− 3.26	44.64	1	44.64	326.39	< 0.0001**
B^2^	− 0.88	3.25	1	3.25	23.77	0.0018**
C^2^	− 2.80	33.04	1	33.04	241.55	< 0.0001**
Residual		0.96	7	0.14		
Lack of fit		0.49	3	0.16	1.39	0.3673
Pure error		0.47	4	0.12		
Core total		118.88	16			

## The response surface methodology analysis

**Fig. 5:**
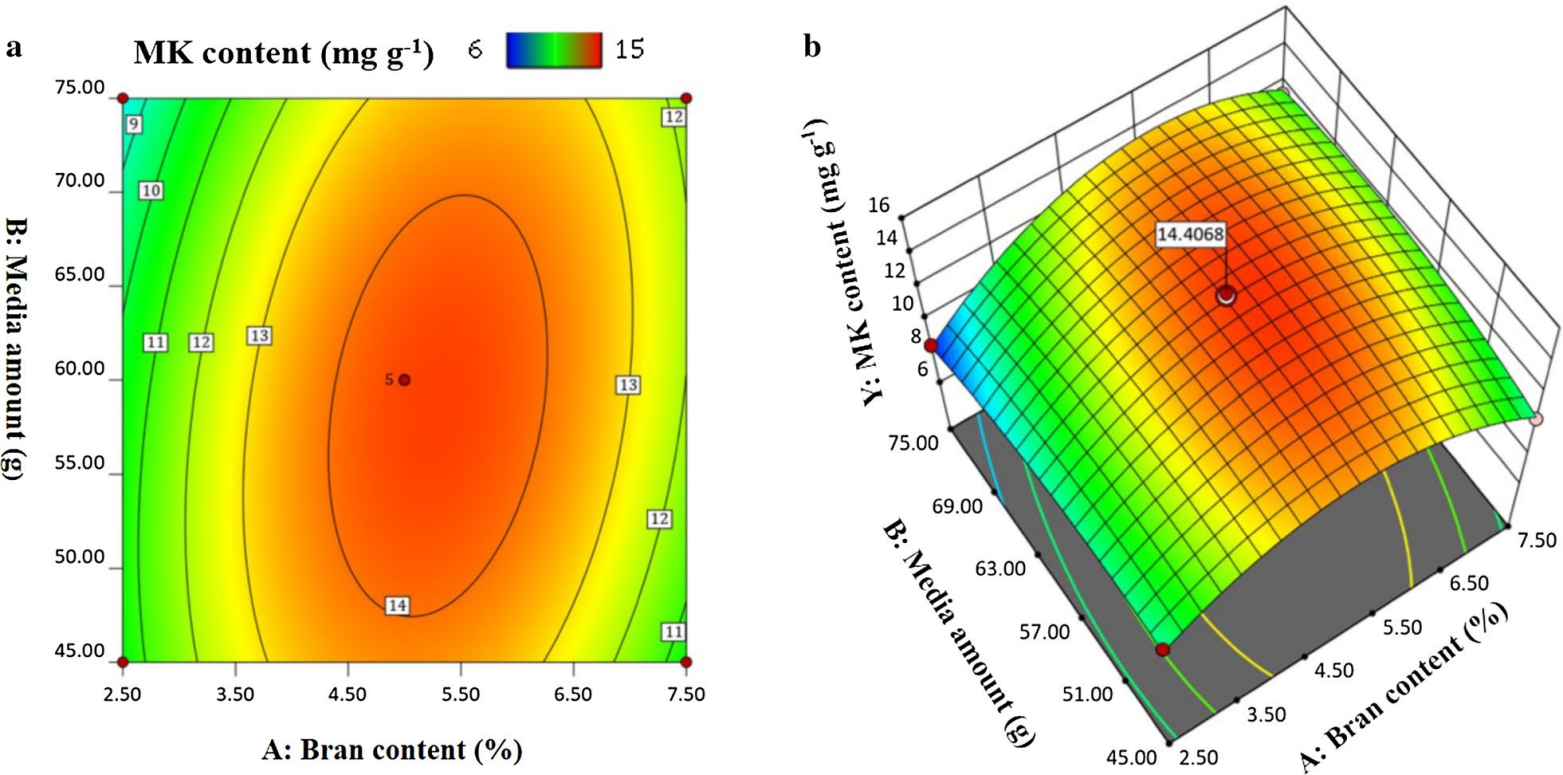
Interaction between bran content and media amount at a fixed initial water of 50%: (a) Contour plot, (b) 3D Surface plot

The results in Fig. 5 showed the MK content decreased significantly at a low bran content ranging from 2.5 to 3.5% and a high media amount ranging from 65 to 75 g. The high media amount was not directly enhanced the MK content and a proper bran content of 4.5 ~ 5.5% was a key factor for MK production. The reason may be the high media amount leads to inadequate oxygen, the addition of bran could improve the oxygen exchange rate, thus enhancing the MK production.

**Fig. 6:**
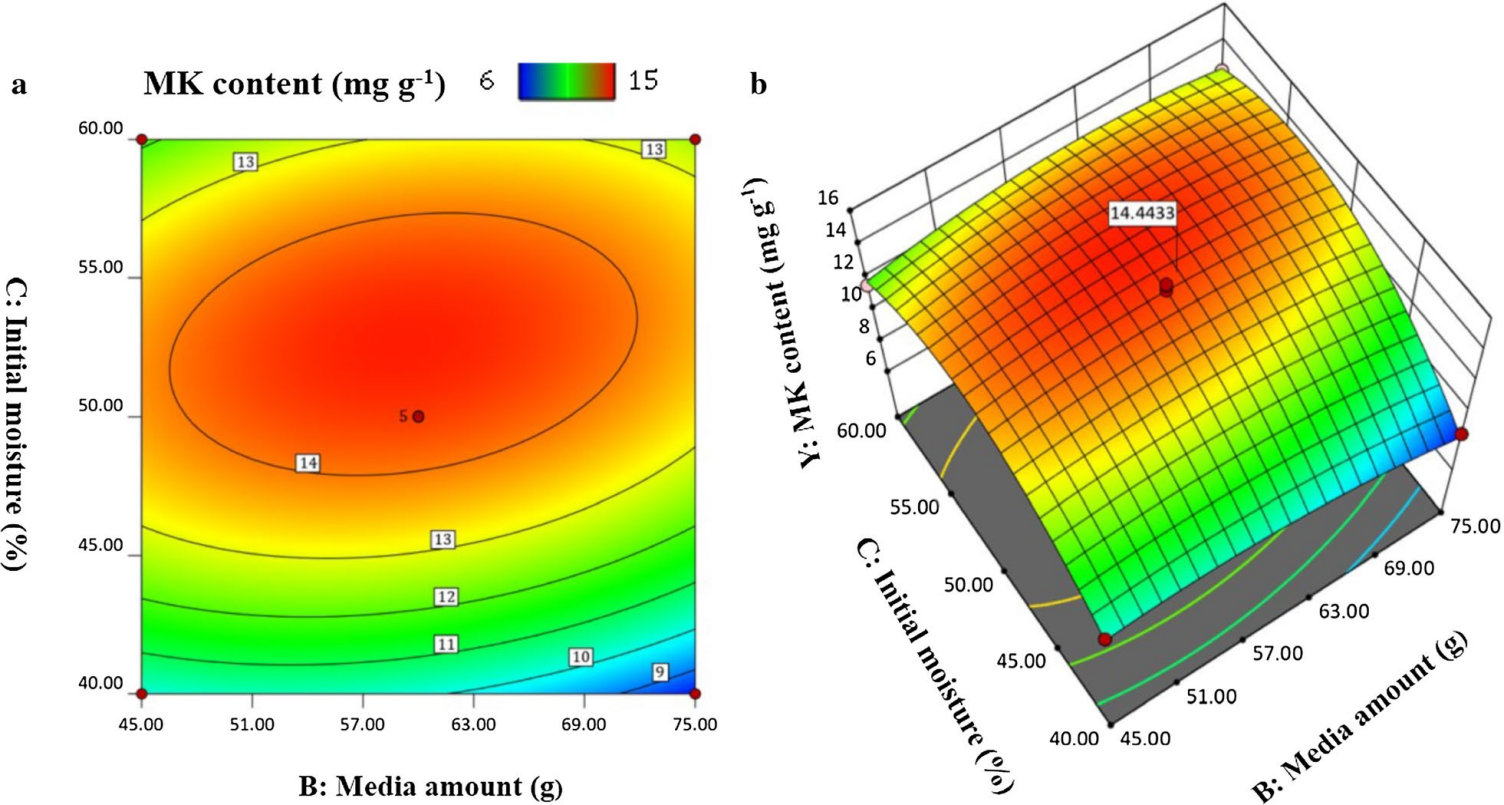
Interaction between media amount and initial moisture at a fixed bran content of 5%: (a) Contour plot and (b) 3D Surface plot

Figure 6 showed the influences of media amount and initial moisture on the production of Monacolin K. The higher MK content was obtained at high initial moisture ranging from 50 to 55%, while the media amount has little influence. The results indicated that water is the important transport medium for nutrients and oxygen during the SSF process of *Monascus*.

**Fig. 7:**
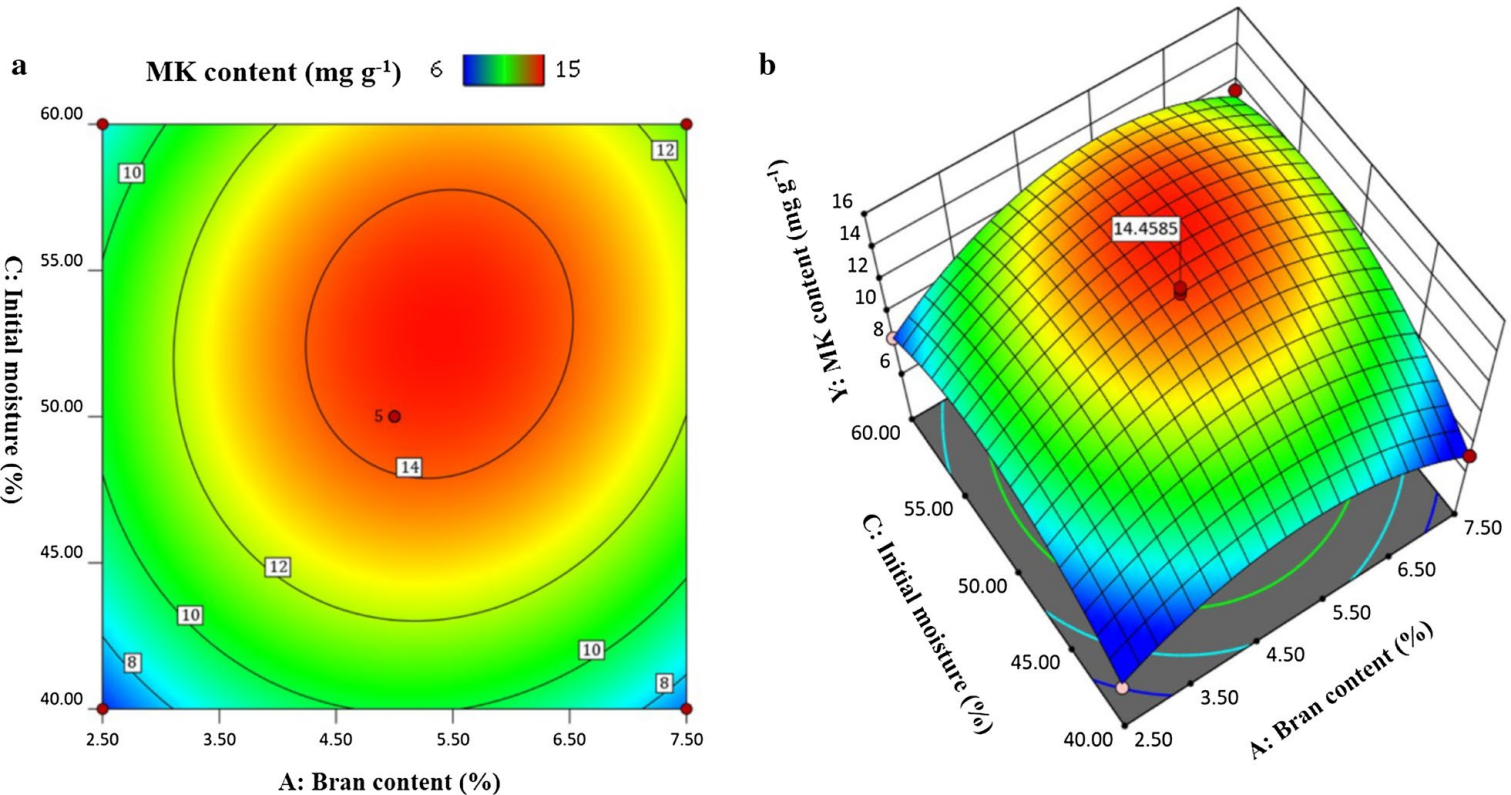
Interaction between bran content and initial moisture at a fixed media amount of 60 g: (a) Contour plot and (b) 3D Surface plot

The results in Fig. 7 showed the minimum yield of Monacolin K was obtained at low initial moisture of 40%, no matter how the bran content changed. The results indicated that MK production could not be adjusted significantly when the initial water is too low. For the industrial production of MK, the initial moisture should be considered firstly.

## Discussion

A detailed study of 7 single-factor experiments and a series of experiments with Plackette-Burman and Box-Benhnken design, data fitting and modeling, and analyzing the visual 3D response surface plots for investigation of the key factors for MK production has been proposed. The results indicated that the initial moisture should be considered firstly during the SSF process. The water performed as a transport medium for nutrients and oxygen exchange, while too high initial moisture would cause the gap in the fermentation medium to become smaller, which was adverse to oxygen utilization (Pansuriya et al. 2010). A proper bran content (4.5 ~ 5.5%) could reduce the impact of too much media amount on MK production, especially useful for the situation of overload media during the industrial production of Monacolin K by *Monascus* (Almeida et al. 2021). The reasons may be a certain amount of bran to the culture medium could improve the permeability of the medium and reduce the free water in the medium, which is beneficial to the production of Monacolin K. For carbon and nitrogen sources, the carbon source with a simpler structure and organic nitrogen source was more beneficial to the growth and metabolism of *Monascus*. Variable temperature cultures are useful for an increase of MK yield, because the relatively high temperature in the early stage of fermentation could increase the number of mycelia rapidly, and the low temperature in the later stage of fermentation is beneficial to the growth and development and MK synthesis (Lin et al. 2017). Under the optimal fermentation conditions of 36 h seed inoculation time, 37.7% of rice content, 3% of glucose content, 1.5% of peptone, 14 mesh of rice grain size, pH value of 5.0, 8% of inoculation amount, 5.39% of bran content, 60.43 g of media amount and 52.86% of initial moisture, the *monascus ruber* was fermented at 30℃ for 3 days and then fermented 26 ℃ for 15 days to obtain a higher MK content of 14.53 ± 0.16 mg·g^− 1^, which was roughly consistent with the predicted value of 14.68 ± 0.37 mg g^− 1^. Table 3 showed the comparison of the present method with recent studies, the results indicated our method has higher efficiency for the MK production by SSF.

**Table 3:** Comparison of the present method with recent studies

Strain	Fermentation parameters	MK production (mg g^-1^)	References
*M. purpureus CMU002UXX-32-44*	Nutrients: NH_4_Cl, MgSO_4_ 7H_2_O	6.24	Kanpiengjai et al. (2018)
*M. buliginosus M1*	Inorganic salts of divalent metal cations	9.57	Lin et al. (2019)
*M. pilosus* 305-9	Amount of added water	0.75	Oh et al. (2021)
*M. purpureus* IF-RPD 4046	initial moisture and white rice	1.33	Saithong et al. (2019)
*M. ruber* M2-1	Chinese medicines	3.6	Peng et al. (2020)
*M. ruber*	Using millet as substrate	7.12	Zhang et al. (2018)
*M. sp.* (BCRC31,615)	Mutation and temperature variation	8.44	Huang et al. (2019)
*M. ruber* (K10403)	Multi-factors	14.54	Our method*

## Supplementary Information

**Additional file 1.** **Figure S1 **HPLC chromatogram chart of Monacolin K; **Figure S2** The standard curve of Monacolin K and **Table S1** Recoveries obtained from the analysis of red yeast rice samples spiked with Monacolin K; **Table S2** Plackett-Burman experiment design for single-factor experiments; **Table S3** Box-Benhnken experiment design for significant factors.

## Data Availability

The datasets generated during and/or analyzed during the current study are available from the corresponding author on reasonable request.

## References

[CR1] Almeida AB, Santos NH, Lima TM, Santana RV, Oliveira Filho JG, Peres DS, Egea MB (2021). Pigment bioproduction by Monascus purpureus using corn bran, a byproduct of the corn industry. Biocatal Agr Biotechnol.

[CR2] Chang YN, Huang JC, Lee CC, Shih IL, Tzeng YM (2002). Use of response surface methodology to optimize culture medium for production of lovastatin by *Monascus ruber*. Enzyme Microb Tech.

[CR3] Dikshit R, Tallapragada P (2016). Statistical optimization of lovastatin and confirmation of nonexistence of citrinin under solid-state fermentation by *Monascus sanguineus*. J Food Drug Anal.

[CR4] Ezhov MV, Catapano A, Escobar C, Kukharchuk VV, Voevoda MI, Drapkina OM, Shalnova SA, Starodubova AV, Gurevich VS, Shaposhnik II, Bolshakova OO, Oynotkinova OS, Alieva AS (2020). The role of red yeast rice based preparations for non-pharmacological correction of dyslipidemia in patients with low and moderate cardiovascular risk (expert opinion). Ration Pharmacother.

[CR5] Handa CL, de Lima FS, Guelfi MFG, Fernandes MDS, Georgetti SR, Ida EI (2019). Parameters of the fermentation of soybean flour by *Monascus purpureus* or *Aspergillus oryzae* on the production of bioactive compounds and antioxidant activity. Food Chem.

[CR6] Huang CF, Shen SM, Chen WT, Chen CC (2019). The effects of mutation and temperature variation on monacolin K production by *monascus sp*. and relative statistical parameter analysis of monacolin K production. Phytochem Lett.

[CR7] Kanpiengjai A, Mahawan R, Pengnoi P, Lumyong S, Khanongnuch C (2018). Improving the monacolin K to citrinin production ratio in red yeast rice by an X-ray-induced mutant strain of *Monascus purpureus*. Bio Technologia.

[CR8] Lee CL, Pan TM (2012). Development of *Monascus* fermentation technology for high hypolipidemic effect. Appl Microbiol Biotechnol.

[CR9] Lin L, Wang CL, Li ZJ, Wu HJ, Chen MH (2017). Effect of temperature-shift and temperature-constant cultivation on the Monacolin K biosynthetic gene cluster expression in *Monascus sp*. Food Technol Biotechnol.

[CR10] Lin L, Jiang L, Guo HZ, Yang L, Liu ZZ (2019). Optimization of divalent metal cations for maximal concentration of Monacolin K in *Monascus* M1 by response surface methodology. Czech J Food Sci.

[CR11] Lu LP, Zhang BB, Xu GR (2013). Efficient conversion of high concentration of glycerol to Monacolin K by solid-state fermentation of *Monascus purpureus* using bagasse as carrier. Bioprocess Biosyst Eng.

[CR12] Mohan-Kumari HP, Dhale MA, Govindaswamy V (2012). Optimazation of Monacolin K production by *Monascus purpureus* MTTC 410 in submerged fermentation. Int J Food Eng.

[CR13] Oh HA, Kim MY, Lee YJ, Lee J, Jeong HS (2021). Effects of amount of added water on red yeast rice production using Korean soft rice variety “Hangaru”. Inter J Food Eng.

[CR14] Panda BP, Javed S, Ali M (2010). Optimization of fermentation parameters for higher lovastatin production in red mold rice through co-culture of *Monascus purpureus* and *Monascus ruber*. Food Bioprocess Tech.

[CR15] Pansuriya RC, Singhal RS (2010). Response surface methodology for optimization of production of lovastatin by solid state fermentation. Braz J Microbiol.

[CR16] Peng L, Ai-Lati A, Liu S, Ji Z, Mao J, Che X (2020). Effects of Chinese medicines on monacolin K production and related genes transcription of *Monascus ruber* in red mold rice fermentation. Food Sci Nutr.

[CR17] Saithong P, Chitisankul WT, Nitipan S (2019). Comparative study of red yeast rice with high monacolin K, low citrinin concentration and pigments in white rice and brown rice. Czech J Food Sci.

[CR18] Subhagar S, Aravindan R, Viruthagiri T (2009). Response surface optimization of mixed substrate solid state fermentation for the production of lovastatin by *Monascus purpureus*. Eng Life Sci.

[CR19] Suh SH, Rheem S, Mah JH, Lee W, Byun MW, Hwang HJ (2007). Optimization of production of monacolin K from gamma-irradiated *Monascus mutant* by use of response surface methodology. J Med Food.

[CR20] Sun JL, Zou X, Liu AY, Xiao TF (2011). Elevated yield of monacolin K in *Monascus purpureus* by fungal elicitor and mutagenesis of UV and LiCl. Biol Res.

[CR21] Suraiya S, Kim JH, Tak JY, Siddique MP, Young CJ, Kim JK, Kong IS (2018). Influences of fermentation parameters on lovastatin production by *Monascus purpureus* using Saccharina japonica as solid fermented substrate. Lwt.

[CR22] Theunis M, Naessens T, Verhoeven V, Hermans N, Apers S (2017). Development and validation of a robust high-performance liquid chromatographic method for the analysis of monacolins in red yeast rice. Food Chem.

[CR23] Wen QY, Cao XH, Chen ZT, Xiong ZX, Liu JH, Cheng ZX, Zheng ZH, Long CN, Zheng BD, Huang ZW (2020). An overview of *Monascus* fermentation processes for monacolin K production. Open Chem.

[CR24] Xiong Z, Cao X, Wen Q, Chen Z, Cheng Z, Huang X, Zhang Y, Long C, Zhang Y, Huang Z (2019). An overview of the bioactivity of monacolin K / lovastatin. Food Chem Toxicol.

[CR25] Zhang BB, Lu LP, Xu GR (2015). Why solid-state fermentation is more advantageous over submerged fermentation for converting high concentration of glycerol into Monacolin K by *Monascus purpureus* 9901: A mechanistic study. J Biotechnol.

[CR26] Zhang C, Liang J, Yang L, Chai S, Zhang C, Sun B, Wang C (2017). Glutamic acid promotes monacolin K production and monacolin K biosynthetic gene cluster expression in *Monascus*. AMB Express.

[CR27] Zhang BB, Xing HB, Jiang BJ, Chen L, Xu GR, Jiang Y, Zhang DY (2018). Using millet as substrate for efficient production of monacolin K by solid-state fermentation of *Monascus ruber*. J Biosci Bioeng.

[CR28] Zhang C, Chai S, Hao S, Zhang A, Zhu Q, Zhang H, Wang C (2019). Effects of glutamic acid on the production of monacolin K in four high-yield monacolin K strains in *Monascus*. Appl Microbiol Biotechnol.

[CR29] Zhou G, Fu L, Li X (2014). Optimisation of ultrasound-assisted extraction conditions for maximal recovery of active monacolins and removal of toxic citrinin from red yeast rice by a full factorial design coupled with response surface methodology. Food Chem.

