# Supplementary material for: A systematic study of the production of Monacolin K by solid state fermentation of Monascus ruber
Source: AMB Express. 2022 Mar 3;12:29. doi: 10.1186/s13568-022-01368-z (PMC8894543; doi:10.1186/s13568-022-01368-z)
Supplement: Supplementary file 1 — Additional file 1. Figure S1 HPLC chromatogram chart of Monacolin K; Figure S2 The standard curve of Monacolin K and Table S1 Recoveries obtained from the analysis of red yeast rice samples spiked with Monacolin K; Table S2 Plackett-Burman experiment design for single-factor experiments; Table S3 Box-Benhnken experiment design for significant factors. [file 13568_2022_1368_MOESM1_ESM.docx]

*AMB Express*

**A systematic study of the production of** **Monacolin K** **by solid state fermentation of *Monascus* *ruber***

Xiuhe Liu^1^, Aonan Sun^1^, Qing Li^1^ Yamin Du^1^ and Tao Zhao^1^*

^1^School of Food Science and Engineering, Qilu University of Technology (Shandong Academy of Sciences), Jinan 250353, P.R. China.

*Correspondence: Tao Zhao, e-mail address: zhaotao1989@126.com, telephone: +86 53189631195.

**HPLC detection method for Monacolin K content**

**^
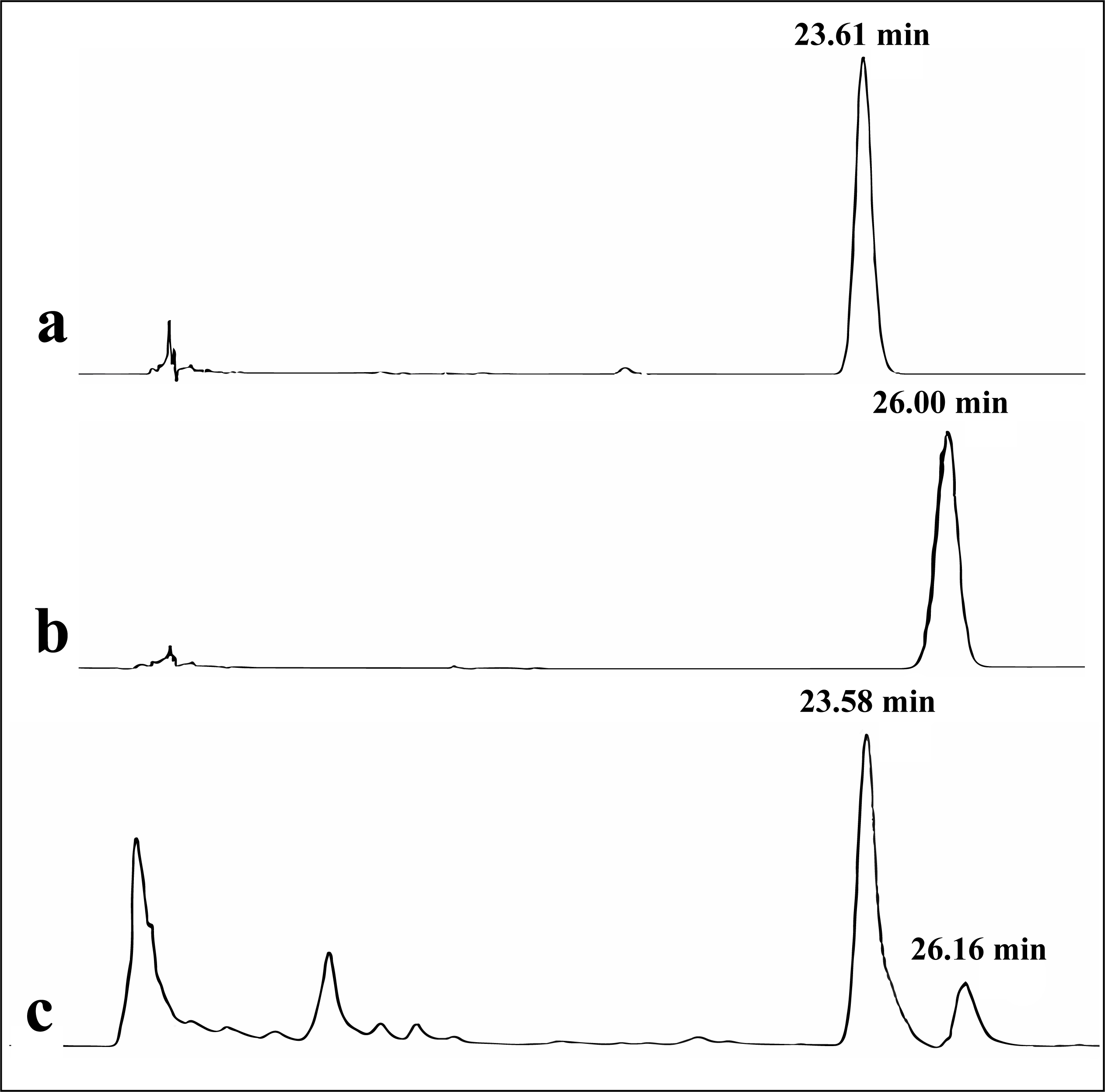
^**

**Figure S1** HPLC chromatogram chart of Monacolin K, (a) acidic Monacolin K standards; (b) lovastatin Monacolin K standards; (c) Monacolin K in red yeast rice.

The formula for calculating the content of monacolin K in the red yeast rice sample

Where, X is the content of monacolin K (mg g^-1^); h_1_ and h_2_ are the peak areas of acidic and lovastatin Monacolin K in the sample; c is the concentration of Monacolin K standard solution (ug mL^-1^); 50 is the constant volume (mL); h_3_ is the peak area of the Monacolin K standard; m is the sample weight (g).


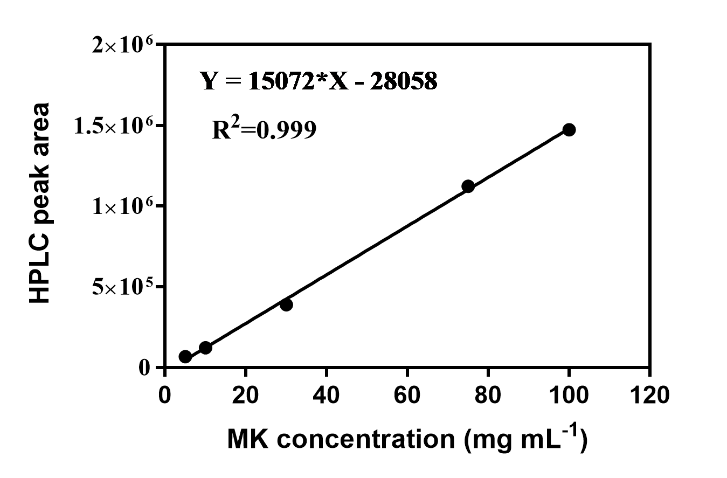


**Figure S2** The standard curve of Monacolin K

| Treatments | Addition content (μg) | Detection content (μg) | Recovery (%) |
| --- | --- | --- | --- |
| 1 | 40 | 38.23 | 95.58 |
| 2 | 80 | 77.45 | 96.81 |
| 3 | 120 | 123.03 | 103.52 |
| 4 | 160 | 156.22 | 97.64 |
| 5 | 200 | 195.30 | 97.65 |
| 6 | 240 | 234.59 | 97.75 |

**Table S1** Recoveries obtained from the analysis of red yeast rice samples spiked with Monacolin K

**Optimization of seed inoculation time**

The seed medium was prepared at 30℃ in a shaker for different culture time. The seed medium was firstly centrifuged at 8000 r min^-1^ for 10 min after cultivation, and then the precipitate was taken out and dried in an oven to constant weight. The growth status of the seed liquid is determined by the weight of the dry seed media.

**

**

**Figure S3** The relationship between culture time and cell dry weight.

The results in Figure S3 showed the relationship between culture time and cell dry weight, the seed started in logarithmic growth phase at 30 h. Therefore, 36 h was set as the best time of inoculation conducive to the production of Monacolin K.

**Table S2** Plackett-Burman experiment design for single-factor experiments

| Run  order | X_1_  (%) | X_2_  (%) | X_3_  (%) | X_4_  (%) | X_5_  (%) | X_6_  (g) | X_7_  (%) | X_8_ | X_9_ | X_10_ | X_11_ | Monacolin K  (mg g^-1^) |
| --- | --- | --- | --- | --- | --- | --- | --- | --- | --- | --- | --- | --- |
| 1 | + (50) | - (3) | + (2) | + (10) | - (4) | + (60) | + (12) | + | - | - | - | 8.32 |
| 2 | + (50) | + (3.5) | - (1) | + (10) | + (6) | + (60) | - (8) | - | - | + | - | 8.49 |
| 3 | - (40) | + (3.5) | + (2) | + (10) | - (4) | - (45) | - (8) | + | - | + | + | 7.31 |
| 4 | + (50) | + (3.5) | - (1) | - (7.5) | - (4) | + (60) | - (8) | + | + | - | + | 10.68 |
| 5 | - (40) | - (3) | - (1) | + (10) | - (4) | + (60) | + (12) | - | + | + | + | 3.68 |
| 6 | - (40) | - (3) | + (2) | - (7.5) | + (6) | + (60) | - (8) | + | + | + | - | 6.84 |
| 7 | - (40) | - (3) | - (1) | - (7.5) | - (4) | - (45) | - (8) | - | - | - | - | 8.79 |
| 8 | + (50) | - (3) | + (2) | + (10) | + (6) | - (45) | - (8) | - | + | - | + | 9.59 |
| 9 | - (40) | + (3.5) | + (2) | - (7.5) | + (6) | + (60) | + (12) | - | - | - | + | 9.99 |
| 10 | - (40) | + (3.5) | - (1) | + (10) | + (6) | - (45) | + (12) | + | + | - | - | 9.24 |
| 11 | + (50) | + (3.5) | + (2) | - (7.5) | - (4) | - (45) | + (12) | - | + | + | - | 13.32 |
| 12 | + (50) | - (3) | - (1) | - (7.5) | + (6) | - (45) | + (12) | + | - | + | + | 12.20 |

**Table S3** Box-Benhnken experiment design for significant factors

| Run numbers | A  (%) | B  (g) | C  (%) | Monacolin K  (mg·g^-1^) |
| --- | --- | --- | --- | --- |
| 1 | -1 (2.5) | 0 (60) | -1 (40) | 6.21 |
| 2 | 0 (5) | 1 (75) | -1 (40) | 8.57 |
| 3 | 0 (5) | 0 (60) | 0 (50) | 14.67 |
| 4 | 0 (5) | 0 (60) | 0 (50) | 14.42 |
| 5 | 1 (7.5) | -1 (45) | 0 (50) | 10.43 |
| 6 | -1 (2.5) | 1 (75) | 0 (50) | 8.45 |
| 7 | 1 (7.5) | 0 (60) | -1 (40) | 7.14 |
| 8 | -1 (2.5) | -1 (45) | 0 (50) | 10.73 |
| 9 | 1 (7.5) | 1 (75) | 0 (50) | 11.45 |
| 10 | 0 (5) | 1 (75) | 1 (60) | 12.47 |
| 11 | 0 (5) | -1 (45) | -1 (40) | 10.21 |
| 12 | 1 (7.5) | 0 (60) | 1 (60) | 11.58 |
| 13 | 0 (5) | 0 (60) | 0 (50) | 13.95 |
| 14 | 0 (5) | 0 (60) | 0 (50) | 14.78 |
| 15 | 0 (5) | -1 (45) | 1 (60) | 11.63 |
| 16 | 0 (5) | 0 (60) | 0 (50) | 14.18 |
| 17 | -1 (2.5) | 0 (60) | 1 (60) | 8.44 |
